# Editorial: New insights into the metabolic processes of immune-related diseases by multi-omics technologies

**DOI:** 10.3389/fendo.2024.1397644

**Published:** 2024-03-26

**Authors:** Yumeng Huang, Huiwen Ren, Yejun Tan

**Affiliations:** ^1^ Department of Endocrinology and Metabolism, Tianjin Medical University General Hospital, Tianjin, China; ^2^ Department of Pharmacology, Tianjin Key Laboratory of Inflammatory Biology, Center for Cardiovascular Diseases, Key Laboratory of Immune Microenvironment and Disease (Ministry of Education), The Province and Ministry Co-sponsored Collaborative Innovation Center for Medical Epigenetics, School of Basic Medical Sciences, Tianjin Medical University, Tianjin, China; ^3^ School of Mathematics, University of Minnesota Twin Cities, Minneapolis, MN, United States; ^4^ Department of Rheumatology and Immunology, The Second Xiangya Hospital of Central South University, Changsha, China

**Keywords:** immunity, auto-immunity, multi-omic, immunometabolism, immune cell activation

## Introduction

The intricate dance between metabolic processes and immune system regulation is pivotal in maintaining health and combating disease (1). However, when this balance is disrupted, it can lead to a cascade of immune-related diseases, ranging from autoimmune disorders to malignancies. Understanding the nuances of this metabolic dysregulation poses a formidable challenge due to the complex interplay of genetic, environmental, and cellular factors. Enter the era of multi-omics technologies—a beacon of hope in this convoluted landscape (2). These cutting-edge tools, encompassing genomics, transcriptomics, proteomics, and metabolomics, have heralded a new age of discovery. They allow researchers to peel back the layers of biological complexity, providing a systems-level view that captures the dynamic interactions between metabolic pathways and immune responses (3). This technological revolution not only offers fresh insights into the molecular underpinnings of immune-related diseases but also paves the way for innovative therapeutic strategies, promising a future where interventions are more targeted, personalized, and effective.

## Aims and objectives

This Research Topic is dedicated to harnessing the power of multi-omics technologies to illuminate the complex metabolic underpinnings of immune-related diseases. Our primary goal is to dissect and understand the intricate metabolic pathways that become dysregulated in such conditions, shedding light on their roles in the pathogenesis and progression of diseases. By leveraging the comprehensive and integrative nature of genomics, transcriptomics, proteomics, and metabolomics, we aim to identify novel metabolic pathways that are pivotal in immune cell function and dysfunction. A key objective is to unravel the functional significance of these pathways in modulating immune cell behavior, such as activation, differentiation, and response to pathogens or malignancies. Ultimately, this endeavor seeks to uncover potential therapeutic targets within these metabolic pathways, offering new avenues for intervention and treatment strategies that could revolutionize the management of immune-related diseases.

## Overview of contributions

The contributions within this Research Topic unveil the multifaceted role of metabolic processes in immune-related diseases through the lens of multi-omics technologies. One study delves into the protective role of alpha linolenic acid metabolism in nasopharyngeal carcinoma, unveiling a prognostic model based on differentially expressed genes associated with this metabolic pathway (Fang et al.). This work not only sheds light on the metabolic underpinnings of tumor progression but also underscores the potential of targeted metabolic interventions in improving patient outcomes.

Another pivotal contribution explores the prognostic significance of basement membrane-related genes in breast cancer, establishing a gene signature that correlates with the tumor immune microenvironment and patient prognosis (Cai et al.). This research exemplifies the intricate relationship between the structural components of the tumor microenvironment and metabolic processes, offering new insights into the mechanisms driving cancer progression and potential therapeutic targets.

Further expanding the scope, a study on glioma stages through a multi-omics approach highlights how alterations in cellular metabolism are closely intertwined with tumor development and grading (Yan et al.). This comprehensive analysis not only enriches our understanding of glioma metabolism but also paves the way for more personalized diagnostic and therapeutic strategies based on metabolic profiling.

In the realm of autoimmune diseases, an investigation employing Mendelian Randomization reveals the causal links between specific serum metabolites and the risk of psoriasis, emphasizing the role of lipid metabolism in the disease’s pathogenesis. This novel approach to understanding the metabolic basis of psoriasis opens avenues for targeted metabolic therapies.

Moreover, the bidirectional association between non-scarring alopecia and hypothyroidism, explored through Mendelian Randomization, highlights the genetic underpinnings and potential metabolic pathways connecting these conditions. This finding suggests broader implications for understanding autoimmune diseases and their metabolic context.

Lastly, the relationship between autoimmune thyroid antibodies and anti-nuclear antibodies offers a glimpse into the complex interactions within the immune system, with implications for diagnosing and managing autoimmune thyroid diseases. This study contributes to a deeper understanding of the autoimmune spectrum and its metabolic associations.

Collectively, these contributions underscore the pivotal role of metabolic processes in immune-related diseases, highlighting the potential of multi-omics technologies to unravel these complex interactions and pointing toward novel therapeutic targets and strategies.

## Implications and future directions

The insights garnered from this collection of studies underscore a paradigm shift in the understanding and management of immune-related diseases, steering us toward an era of personalized medicine. The elucidation of metabolic pathways and their interplay with the immune system opens new vistas for disease diagnosis and monitoring. By pinpointing specific metabolic dysregulations, clinicians can potentially diagnose diseases earlier and with greater precision, tailoring treatments to the individual’s unique metabolic profile.

Moreover, the identification of novel therapeutic targets within these metabolic pathways promises to refine treatment strategies, moving beyond one-size-fits-all approaches to interventions that are meticulously aligned with the patient’s specific disease mechanisms. This could not only enhance the efficacy of treatments but also reduce adverse effects, significantly improving patient outcomes.

However, these pioneering findings are just the tip of the iceberg. To translate this wealth of knowledge into clinical practice, further research is imperative. Future studies should focus on validating these metabolic targets in diverse populations, exploring the long-term effects of targeting these pathways, and integrating multi-omics data with clinical observations. Such endeavors will pave the way for truly personalized healthcare, transforming the prognosis for individuals afflicted with immune-related diseases.

## Call to action

We call upon the global scientific community to forge ahead in this exciting frontier, embracing collaboration and interdisciplinary synergy. Let us remain steadfast in our pursuit of innovation, delving deeper into the metabolic-immune nexus to unveil novel solutions that hold the promise of a healthier future for all.

## Author contributions

YT: Writing – original draft, Writing – review & editing. YH: Writing – original draft, Writing – review & editing. HR: Writing – original draft, Writing – review & editing.
